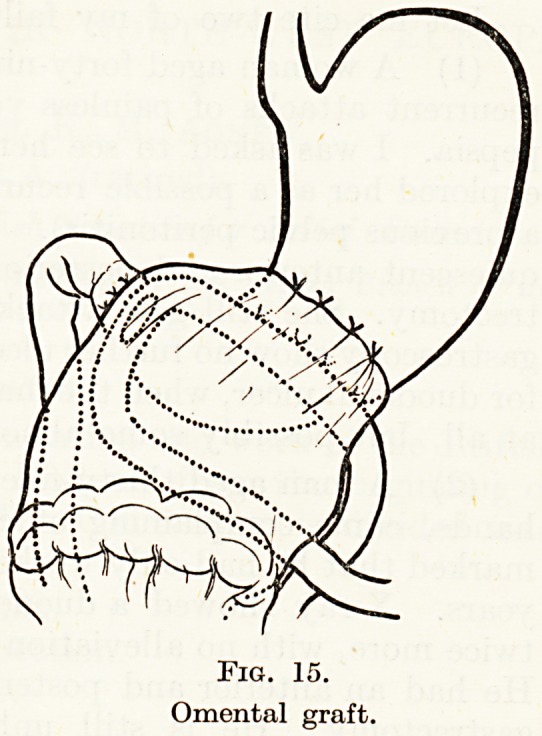# The Management of Peptic Ulcer

**Published:** 1946

**Authors:** Norman C. Tanner

**Affiliations:** Senior Surgeon, St. James's Hospital, London; Surgeon Specialist to the London County Council


### THE MANAGEMENT OF PEPTIC ULCER

BY

NORMAN C. TANNER, M.B., CH.B., F.R.C.S.

*Senior Surgeon, St. James's Hospital, London ;*
*Surgeon Specialist to the London County Council.*

A paper read at a Meeting of the Bristol Medico-Chirurgical Society on Wednesday, November 14th, 1945.

THE management of peptic ulcer is a big subject to discuss exhaustively, and so I will deal with three aspects of it—firstly, the significance of periodicity ; secondly, the factors producing healing of the ulcer ; and thirdly, the surgical aspect.

Let me begin by reviewing some of the events in the life of a sufferer from this disease. The patient, usually an otherwise healthy person in youth or middle age, gets an attack of discomfort or pain associated with meals. He ascribes it to some recent business worry, cold, or dietetic indiscretion. Discussion with friends introduces him to the use of alkali—which he finds gives him temporary relief—and after the use of several patent brands the pain leaves him and the last brand he used is given the credit. Later on—days, weeks, months or even years after—another attack occurs, and this is followed by fresh attacks. Again the attacks are attributed to some antecedent event, and the patient treats himself with diet and alkalies, or may seek medical advice. Each time he gets a varying degree of relief for which he thanks his diet, his powder or his doctor. So the attacks tend to go on, relapse and remission following each other, each with its facile explanation.

The completeness of the relief is such that there is delay in seeking medical advice, and the patient is averse to having treatment which keeps him from his work during times of comfort. Even if he is forced to his bed by an attack, the completeness of the relief which follows makes him unwilling to remain there long.

Of course we all know that this feature of periodical remission and relapse—periodicity—is typical of gastric and duodenal ulceration. It is also the curse of the disease, for the patient again and again is lulled into a false sense of security by the remissions. For the pain goes before the ulcer is healed, or even while it is active, and the patient returns to his work, or to the practices which caused the ulcer to appear, and this goes on and on till such extensive damage is done to the stomach or duodenum that hope of complete anatomical repair is lost. The periodicity does not necessarily mean alternate healing and breaking down of the ulcer, for radiological or gastroscopic examination during the remissions often shows the presence of active ulceration. Why then does the ulcer suddenly become symptom-free? This question is undecided, and rests on the also unknown answer to the question of the cause of ulcer-pain. In this matter I strongly believe that chemical or acid stimulation of the ulcer is the cause of the pain. This belief is strongly supported by Palmer's test. Palmer's test is to introduce into the stomach 100-200 c.cm. of 0.5 per cent. hydrochloric acid solution, i.e. about the same strength as gastric juice: and in a few minutes, if the patient has an ulcer, he will get pain—and this pain can be relieved by removing the acid, or neutralizing it with alkali. This test may be negative during remissions, even if a gastric ulcer is present.

Similarly others have produced pain in duodenal ulcers by the injection of acid into the duodenum. I have, under local anaesthesia, with only the abdominal wall anaesthetized, exposed a gastric ulcer after opening the stomach and put a swab soaked in decinormal hydrochloric acid into the ulcer, and within 1½ minutes produced pain like ulcer-pain. This was relieved by removal of the swab and replacing it by saline.

If acid stimulation be the cause of pain, then the remissions may result from the nerve-endings being protected by a thick tenacious plug of mucus or by new mucosa growing from the edge. Or possibly a little fresh necrosis of the edge or base might cause temporary death of exposed nerve-endings, and pain would cease until the slough had separated to expose live tissue again. Symptoms are known to cease abruptly after a haematemesis: in such cases one may at times find the crater filled with blood-clot, which would act as a barrier.

Against these theories is the fact that stimulation of the crater with acid during a period of remission of symptoms may not produce pain even if the mucus is scraped out. Possibly inflammation sensitizes the nerve endings in the crater to stimulation.

However, sometimes the remission does mean that the ulcer is healed, but the first main fact we must face in estimating the result of our ulcer treatment is that cessation of pain and other symptoms does not by any means prove that the ulcer has been cured or even improved. Of course, continuance of symptoms suggests that the treatment is probably not succeeding. Unfortunately legions of remedies, injections of foreign protein, mucosal extracts, histidine, endocrines, etc., have had an undeservedly long vogue as a result of the failure to take into account the natural remissions of the disease. Many things appear to produce a remission. Perhaps the most certain methods are to take a holiday, or a week-end in bed, or be admitted to hospital. I recollect once taking a French physician round my wards. He remarked that at his hospital all peptic ulcer cases were admitted to hospital and given a course of injections of protein, and within three days they were free of pain. I was able to shew him that my patients, during this time of bed-shortage, were admitted for a barium meal X-ray the first day, a fractional test meal the second day, and by the time they were due for gastroscopy on the third day their pain was usually gone! This is despite the fact that the examination might shew active lesions.

How then can we judge the efficiency of our treatment if symptoms are too erratic to be of value ? What of physical signs ? These are notably absent except in the presence of a local peritonitis, abscess, deep penetration, or pyloric stenosis. Hyperalgesia of the skin is variable, in true ulcer cases often absent, and very pronounced in the nervous dyspepsias. High epigastric tenderness is more marked in nervous dyspeptics than in cases of true ulcer, and there may be no tenderness when one palpates over an active ulcer. This is not very surprising, for it is possible, after opening the abdomen under local anaesthesia, to palpate an ulcer of the stomach, to pinch or squeeze it, without causing pain.

No: symptoms and physical signs are both poor guides to show the result of our treatment, and only by radiological or gastroscopic examination can we be certain that we have improved or cured the ulcer.

### Factors Producing Healing of the Ulcer

Now supposing that we have discovered that a certain régime produces healing of the ulcer, we should next try to discover what is the most important factor in the régime. It has been my habit to treat ulcer patients on routine lines in hospital, with a bland diet including frequent feeds of milk, alkalies, atropine and bed-rest. Before the war I used the régime recommended by the late Sir Arthur Hurst. Under this régime I was impressed by the rapidity with which acute and subacute and even some chronic gastric ulcers would heal, or at any rate become much smaller : indeed if there was no change after a fortnight's treatment one was very suspicious of malignancy.

However, in an endeavour to find out the most important factor in treatment, and with gastroscopic control, I tried the effect of abandoning one factor after another in patients with gastric ulcers. First I put them on three big meals a day with nothing between meals, the meals being ordinary hospital full diet. There was no change in the rate of healing! Next I abandoned the use of alkalies and atropine : again the ulcers healed at their usual speed. On the other hand, I have tried allowing the patients to go home, on a strict ulcer diet and alkalies, allowing them to work only half-days, and have found that healing is usually slower and uncertain. Thus it appears that bed-rest is the major factor in the healing of these gastric ulcers. Why is this ? Is it some psychological influence, or is it recumbency?

It is fashionable to stress psychological influences, and to suggest that mental rest is the healing factor. We know that relapses are often attributed to business worries and family crises. The effect of air-raids has been cited as evidence of psychological damage affecting peptic ulcers. In Bristol and London medical students have given statistical evidence to show the effect of air-raids on the incidence of perforation of ulcers. You will be interested to see how the fly-bomb raids affected the number of admissions of cases of haematemesis (Fig. 1). In fact, air raids even increased my operative mortality for gastrectomy (Fig. 2).

FIG. 1.

Monthly incidences and mortality of gastroduodenal hæmorrhage (excluding carcinoma and hepatic cirrhosis).

However, all these things do not prove a pure nervous stimulus to ulceration. For emotional upsets, business worries, air-raids and shelter-life mean disordered meal-times, missed meals and missed sleep, and these are physical factors which I believe of great importance in the production of ulceration. Furthermore, bed-rest in hospital does not mean freedom from worry, for many are in grave economic difficulties with their illnesses. A patient was admitted and gastroscopy shewed an ulcer. His employers told him that if he was not fit for work in two weeks he would lose his job. He was agitated, and wanted to leave hospital; on my advice he changed his mind, and a second examination after two weeks (the day

FIG. 2.
Mortality for gastrectomy for innocent ulcer.

before he was to lose his job unless he left hospital) showed perfect healing. However, one must concede that in hospital there is a certain lack of nervous tension which may be present in some occupations, and one must give due weight to psycho-somatic factors.

### THE INFLUENCE OF RECUMBENCY

To discover if the position of the body had any effect I treated patients in Fowler, Trendelenberg and flat positions. Of course there was a tendency for all to get into the flat position, for those in the Fowler slipped down at nights. Also it is difficult to compare rates of healing: ulcers are so variable in their rate of healing even under similar circumstances. To make the comparison a little more accurate, I have also added the average age and average length of history of each group. This was the result, but it must be taken with some reserve :—

| | |
|---|---|
| GASTRIC ULCERS treated first six months, 1943 | 108 |
| Number repeatedly examined in hospital to ascertain time required for healing reasonably accurately .. .. .. | 49 |
| *Flat in Bed.* 15 cases. | |
| Average age .. .. .. .. .. | 53 years |
| Average length of history .. .. .. | 8.6 years |
| Average time required for healing .. .. | 34 days |
| *Fowler position.* 23 cases. | |
| Average age .. .. .. .. .. | 54 years |
| Average length of history .. .. .. | 7.4 years |
| Average time required for healing .. | 49 days |
| *Trendelenberg position.* 11 cases. | |
| Average age .. .. .. .. .. | 55 years |
| Average length of history .. .. .. | 10 years |
| Average time required for healing .. | 35 days |

The average age in each group is about the same, but the Trendelenberg group had the longest history, and so it would appear that the Trendelenberg favourably influences ulcer-healing, while the Fowler position appears to retard it. My clinical impression was that the Trendelenberg position improved healing, and at least one ulcer in a man of 71, which had failed to heal two years in succession, healed on the third attempt in the Trendelenberg position.

There are several factors connected with recumbency which might influence healing.

1. The diminished gastric activity as a result of the diminished nutritional needs of a resting patient. This is unlikely to be a major factor.

2. Some would liken the ulcer to a varicose one, with venous stasis, but there is no evidence of venous stasis and, to say the least, it is a highly unlikely explanation.

3. Cole claims to show a diminished blood-supply to the lesser curvature in the upright position.

4. Diminished tension on the ulcer edges. Normally after taking a meal the stomach increases in size by the slipping over each other of the muscle-fibres and their active elongation, so that though the stomach may descend to the pelvis there is no increase of tension (Fig. 3). In the neighbourhood of an ulcer this process would be hampered, and there might after a meal be some gravitational pull on the edges of the ulcer tending to draw them apart: or if the ulcer was a penetrating one fixed to a neighbouring organ, then the ulcer itself would become one of the points from which the stomach was suspended, so increasing the distractive tension on the ulcer (Fig. 4).

FIG. 3.
Descent of normal stomach during filling.

FIG. 4.
Gastric ulcer, with adhesions fixing affected part of stomach: tension on ulcer when stomach fills.

This would be important because the main healing of an ulcer takes place by contraction of its base and approximation of the edges. (Taking small frequent feeds would have a similar result to recumbency in diminishing gravitational effects).

5. The fifth possibility is that recumbency may change the extent of the chemical stimulus to the ulcer. The body and fundus of the stomach secrete acid gastric juice, the pyloric antrum a feebly alkaline digestive juice (Fig. 5). Also, after eating, the lower part

FIG. 5.
Reactions of gastric secretions.

FIG. 6.
Upright posture: strongly acid secretion in contact with ulcer.

FIG. 7.
Lying down: change in area and position of upper level of fluid.

of the body first fills and then the level gradually rises. The body and fundus act as a reservoir and in the antrum active mixing takes place. In the body there is not much mixing, but according to Cannon a stratification of the food. That is to say the apple pie and custard lie above the roast beef and Yorkshire pudding for some time. Waves of peristalsis in the body may not greatly mix the upper with the lower layers for a time. Now though the whole body is secreting gastric juice, that from the fundus will run down and tend to produce a level of greater concentration on the upper level of the food, so that at a certain stage of digestion, an ulcer in the lesser curvature will have this strong layer of concentrated juice opposite it for a time, and acid juice will pour over it till the end of digestion (Fig. 6). Now the effect of recumbency is to shift the fluid level (Fig. 7)—if the patient lies on the right a lesser curve ulcer will lie under food till nearly the end of digestion ; if he lies on his back most posterior ulcers will be a long time covered by food and also the top level of gastric juice will be wider and so thinner and also will more readily mix with the alkaline pyloric juice and food, so diminishing the acid chemical stimulation to the ulcer. Some of these explanations are rather " far-fetched " ; certainly the subject is a difficult one.

After this digression, back to our main point. We must not forget the strong tendency for the less chronic ulcers to heal when the patient is kept in bed, and we must bear this in mind when we are judging the effects of any new method of treatment which entails bed-rest.

### EARLY TREATMENT OF ULCER DYSPEPSIA

What then should be the procedure when a patient comes to his general practitioner with an ulcer-like dyspepsia for the first time ? If the patient is over thirty-five he should have an X-ray of the stomach and duodenum to exclude carcinoma. If he is youthful he should not be sent away with a diet and some alkali. Such a procedure will satisfy him and probably provoke a remission, but it will also set him on the road to chronicity and the surgeon. He should instead have two to three weeks' rest in bed to cure his ulcer. This is usually enough if it is his first attack : and then he should have his eating habits and his job reviewed, any irregularities or bad habits being corrected before returning him to work. If he fails to get complete relief of pain, then one would suspect extra-gastric or functional dyspepsia or carcinoma, and he should have a radiological examination. If he relapses he should have radiological control of his next course of treatment.

What of alkalies ? Though I have abandoned them experimentally, I use them in practice. It is certainly rational to do so, and I believe there is even a place for the prolonged use of the non-absorbable alkalies, e.g. magnesium trisilicate, in the prevention of relapse.

### THE PLACE OF SURGERY

What part must surgery play in the management of peptic ulceration ? Unfortunately, with our imperfect co-ordination of ulcer treatment, with the great delays which occur before the first satisfactory course is given and the sending of our healed patients back to the conditions which produced the ulcer originally, and particularly the great tendency of the chronic ulcer to relapse even after healing, the part played by surgery must be a large one, quite apart from its place in the treatment of complications such as perforation, abscess-formation or haematemesis.

As surgeons, I think we discover better than physicians, and better even than morbid anatomists and radiologists, how great is the destruction produced by peptic ulceration. When the chronic deep ulcer heals it does so largely by contraction of the ulcer base and approximation of the ulcer edges—thereby narrowing the length and the circumference of the stomach or duodenum. There is also an ingrowth of new columnar epithelium from the edges, but this is often a feeble growth. With years of recurrent healing or partial healing and relapse, more and more normal mucosa is lost until the

FIG. 8.
Deformity of stomach produced by contraction during healing of ulcer.

FIG. 9.
Stenosis of duodenum due to completely encircling ring of ulceration.

Fig. 10.
Pre-stenotic diverticulum.

deficiency produces serious deformity (Fig. 8). In the early stages only a few radiating folds appear, but later on, especially in the duodenum, a stage arises where any further contraction of the base will produce such stenosis as to be incompatible with life. In addition there are in the cases which come to surgery usually more than one duodenal ulcer, often anterior and posterior, and they may coalesce till there is a complete ring of ulceration (Fig. 9).

In such cases I have twice, during a gastrectomy, found that after division of the adhesion around the duodenum it fell in two, because there was a complete circle of completely penetrating ulcer. In most cases of duodenal ulcer that require surgery such a degree of narrowing has occurred that there is dilatation or ballooning of the duodenal wall between pylorus and ulcer, producing the so-called "pre-stenotic diverticulum," (Fig. 10) in itself of no great importance. But these organic changes show how futile is the sufferer's hope of gaining comfort and security from further medical treatment. He really needs a graft of new duodenal tissue to replace that which is lost, and that nature will not supply. Again, in the stomach, an ulcer which appears to be only an inch or so in diameter may really represent the loss of the whole posterior or anterior wall or more, but by vigorous contraction of the base it has become small (Fig. 11).

Fig. 11.
Wide extent of an apparently small ulcer.

Apart from the great anatomical destruction, occasionally an ulcer will not heal because of the rigidity of its base, or fixation of its fibrotic edges to the pancreas or liver. In such cases there is probably no disagreement that surgery is the only means of attaining comfort.

However, I will freely admit that I often perform gastrectomy for ulcers that would in time heal medically. In 1938 we gastroscoped a man who had had a severe haematemesis shortly before. He had been in my ward on two previous occasions with a haematemesis due to gastric ulcer, and each time had healed well. The ulcer was seen to be soundly healed. A colleague, however, jestingly remarked : " Now he is fit, the healed ulcer will make an easy and safe operation ; we should do a gastrectomy." We did not, of course, and the following winter he came in again and died after his haematemesis. There is no doubt that recurrent ulceration is often an indication for surgery. In fact I would suggest that in the decision whether to operate or not, the patient's history is just as important as the present condition of his ulcer. If he has a large ulcer now and only has a three months' history, medical treatment may provide him with a permanent cure. If he has a small ulcer now and gives a history of having had it treated on and off for ten years, you may heal it again, but healing will not be permanent and he will probably be better off with surgical treatment.

There are other factors to take into account when deciding whether to operate or to continue with medical treatment. Age diminishes the hope of medical cure, economic difficulties make repeated and prolonged medical treatment more difficult, weak-willed patients are often incapable of following a post-ulcer régime.

### The Nature of the Surgical Procedure

One may briefly consider the type of surgical procedure. As regards gastric ulcer, I believe there is no real alternative to gastrectomy : if it is very high a Pauchet type of operation, removing a tongue-shaped segment of the lesser curve containing the ulcer, gives excellent results.

Duodenal ulceration provides the chief problem in settling the type of procedure to be followed. While no operation which entails anastomosing stomach to jejunum can be considered free from the risk of recurrent ulceration, there is, I think, no doubt that a high partial gastrectomy is the procedure least likely to be followed by recurrent ulcer and is the best we can do for most patients. However, in later life there is a tendency for the gastric acidity to diminish. Therefore short circuit may be justified in the elderly if the fractional test-meal shows a normal or low acidity or gastroscopy shows a thinning gastric mucosa. Every textbook puts down pyloric stenosis as an indication for gastro-jejunostomy. While a long-standing stenosis may produce a chronic gastritis and lower the gastric acidity, stenosis by no means gives any guarantee of safety. Some of the worst cases of anastomotic ulceration I have seen have followed a short-circuit for stenosed duodenal ulcer, particularly in the young. Gastro-jejunostomy is permissible as a temporary expedient in the poor risk case. A man aged twenty-six was admitted under my care with tetany and severe alkalosis, due to pyloric stenosis, with a blood urea of 216 mgm. The physician considered him unfit for surgery even after prolonged gastric lavage and bed rest. However, I did a short circuit under local anaesthesia and warned the patient to return to me in six months for a second stage. He came back to me eighteen months later in excellent general condition, and his blood urea was normal, but he had a return of discomfort after food for two or three weeks. I operated on him again, and found a very deeply penetrating gastro-jejunal ulcer, for which I performed a high gastrectomy, and he has remained well since. Here the division into two stages made a poor-risk case successful, but as so often happens, he did not appear for the second stage until recurrent symptoms frightened him in.

How we all wish that a gastro-jejunostomy could be made safe from recurrent ulceration. Somerville has recommended a ligation of the gastric vessels at the same time. I have tried this on a few elderly patients, but 20 per cent. of them developed anastomotic ulceration. I must admit, however, since hearing Somerville, that my ligation of the vessels was not so radical as his. There is something to be said for combining gastro-jejunostomy with left subdiaphragmatic vagotomy, which will diminish gastric secretion for about a year, plus removing a part of the acid-secreting body of the stomach, though I have not practised these operations enough to give any personal results.

There are to my mind two serious defects in the posterior gastro-jejunostomy as it is usually done, in the lowest part of the stomach (Fig. 12). This means that the anastomosis is to the acid-secreting part of the body, and that all the time acid-secreting mucosa is adjacent to jejunal mucosa. In addition, acid juice secreted between

FIG. 12.
Usual (low) posterior gastro-jejunostomy.

FIG. 13.
Juxta-pyloric posterior gastro-jejunostomy.

meals and at night tends to collect in the lower part of the body and so bathes the stoma.

It seems to me more reasonable to place the anastomosis in the pyloric antrum : at first I made a posterior juxtapyloric anastomosis (Fig. 13). This is technically difficult, and I later made a number of anterior ones in the same situation (Fig. 14). Now the resting juice lies away from the stoma, and the mucosa stitched to the jejunum is alkali-secreting. I complete the operation by covering the anastomosis with the omentum (Fig. 15). The results over two years have been satisfactory, but one must wait a long time before one can recommend such a method to others.

Now from the patient's point of view, his first and chief desire will be that he should survive the operation, and so I believe that surgery should never be undertaken unless the patient has had a

FIG. 14.
Anterior juxta-pyloric gastro-jejunostomy.

FIG. 15.
Omental graft.

good pre-operative course of bed-rest, full vitamin and high-protein diet, so that he shall be in as good physical condition as possible and his ulcer fairly quiescent. If the patient is too impatient to have this, he had best forego the operation. My graph of the effect of air-raids on mortality shows that other things than surgical skill are involved.

You may be interested in my own figures for gastrectomy for simple ulcer since 1941, excluding operations done purely to arrest haematemesis.

Four years June, 1941—June, 1945. Gastrectomies for simple ulcer (excluding operations purely for the arrest of haemorrhage, .. 393

| | *No. of gastrectomies* | *Died* | *Operative mortality* |
|---|---|---|---|
| Duodenal ulcer .. | 160 | 1 | 0.6% |
| Gastric ulcer .. | 195 | 7 | 3.5% |
| Gastro-jejunal ulcer | 30 | 0 | 0% |

When abdominal surgeons get together the question often arises : Do you ever get patients with prolonged discomforts after gastrectomy—attacks of vomiting, particularly of bile, nausea, flushes, vertigo, faintness ? Now it is not uncommon to have minor disorders of this sort for up to six months after gastrectomy, though usually the more the operation was required the less prominent they are. However, one of the great snags of ulcer-surgery is that both peptic ulcer and functional nervous dyspepsia are common illnesses, and when they are combined they are liable to get operated on, because they prove to be medical failures and the physician is glad to be rid of them.

Let me cite two of my failures :—

(1) A woman aged forty-nine was admitted with prolonged and recurrent attacks of painless vomiting, and a history of mild dyspepsia. I was asked to see her after a ten days' attack and later explored her as a possible recurrent mild obstruction (she had had a previous pelvic peritonitis). Unfortunately I found she had small quiescent anterior and posterior duodenal ulcers, and I did a gastrectomy. She still gets attacks of vomiting although X-rays and gastroscopy show no further ulceration. My mistake was to operate for duodenal ulcer, when the main symptoms were not those of ulcer at all, but possibly some abnormal functional reverse peristalsis.

(2) A man aged thirty-five, thin, morose and with cold clammy hands, came complaining of continuous epigastric discomfort so marked that he had only worked for three months in the last four years. X-ray showed a duodenal ulcer. I treated him medically twice more, with no alleviation of his distress, and finally operated. He had an anterior and posterior inactive duodenal ulcer. I did a gastrectomy. He is still unhappy, and has similar pains and discomforts as before, though he is working with the aid of intensive psychotherapy. Here my mistake was to operate on a man who was "constitutionally inadequate," or, if you like, a functional dyspepsia was his main trouble and his peptic ulcers were comparatively of minor importance. A non-obstructive ulcer does not prevent a young man from working for three years and nine months out of four years. Such a history, and the lack of periods of freedom from pain should have made me doubt whether the duodenal ulcer was really his basic complaint.

I think these two cases will explain the cause of a large percentage of our failures. In such cases with a duodenal ulcer and functional nervous dyspepsia I have tried pyloroplasty, dividing the vagus nerve, and closing the abdomen without doing anything at all : but they all continue to complain. For surgery will never cure the functional dyspepsias, even if there is a coincident organic lesion. In other words, let surgeons be wary of the patient with multiple and persistent symptoms and the quiescent ulcer.

REFERENCES

1. With reference to the hæmatemesis chart : the highest point on the chart—20 cases per month—was that worked out for June, 1944. The fly bombs started June 16th, and the evacuation of London started then and continued for two months or so.

*Deaths from bleeding* increased between December, 1943 and May, 1944 (that was another matter) : for that period I had experimentally started operating on all severe cases of hæmatemesis (a note on this matter is in Gordon Taylor's article on hæmatemesis in the last *B.J.S.*). Of course, the invasion of Europe started early in June and that may have played some part in the raise incidence of bleeding.

2. *Graph on influence of recumbency.*—I realize that this table is not very conclusive, but the interest appears to be the definitely longer time taken by cases in the Fowler position (which is the nearest to the ambulant position), compared with the other two. The slightly increased chronicity of the Trendelenberg group shows that at least it is not worse than in those treated flat. My main endeavour was to differentiate between flat and Fowler—and Trendelenberg was added in an attempt to accentuate the difference.

## Figures and Tables

**Fig. 1. f1:**
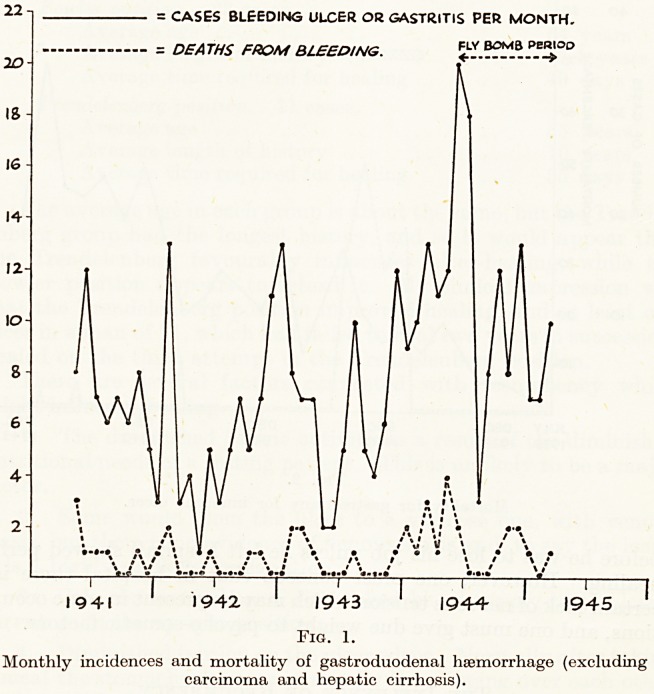


**Fig. 2. f2:**
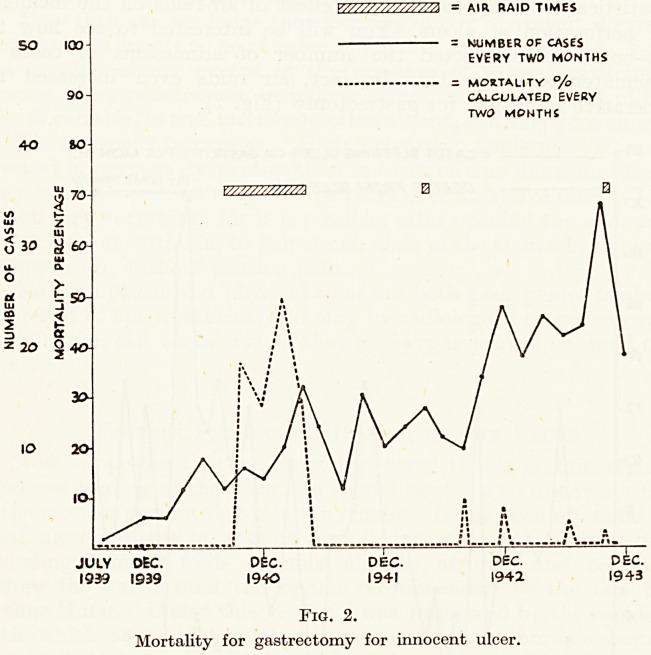


**Fig. 3. f3:**
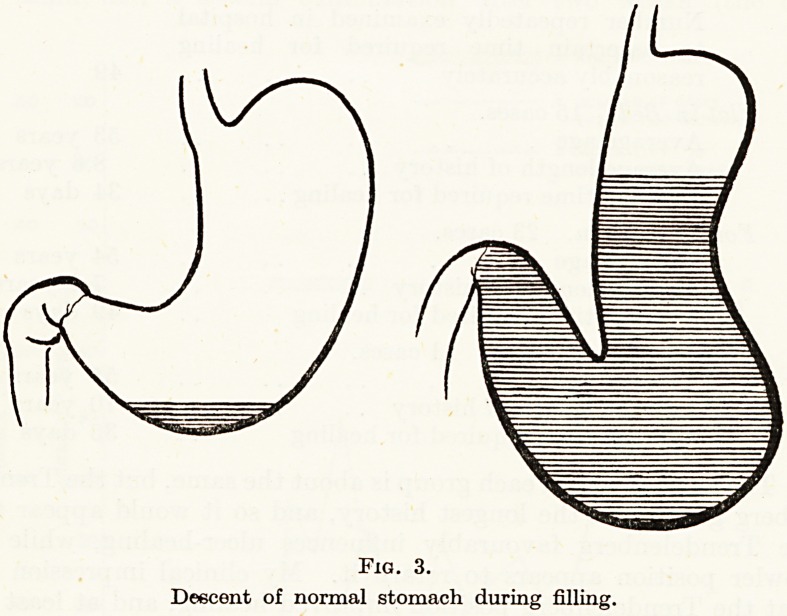


**Fig. 4. f4:**
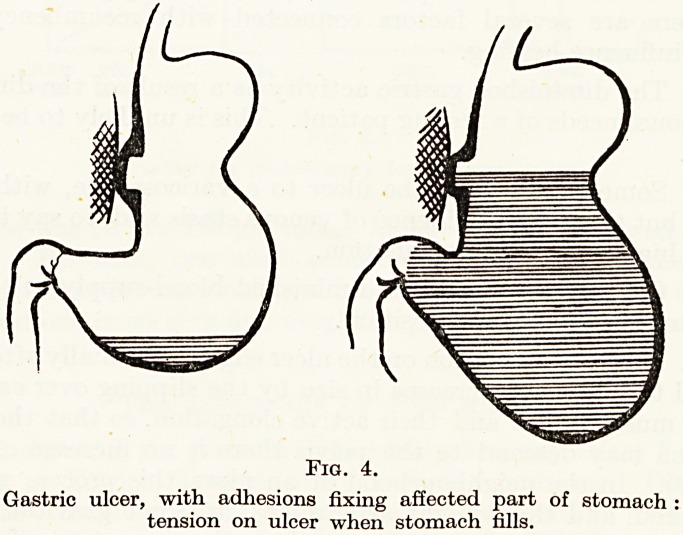


**Fig. 5. f5:**
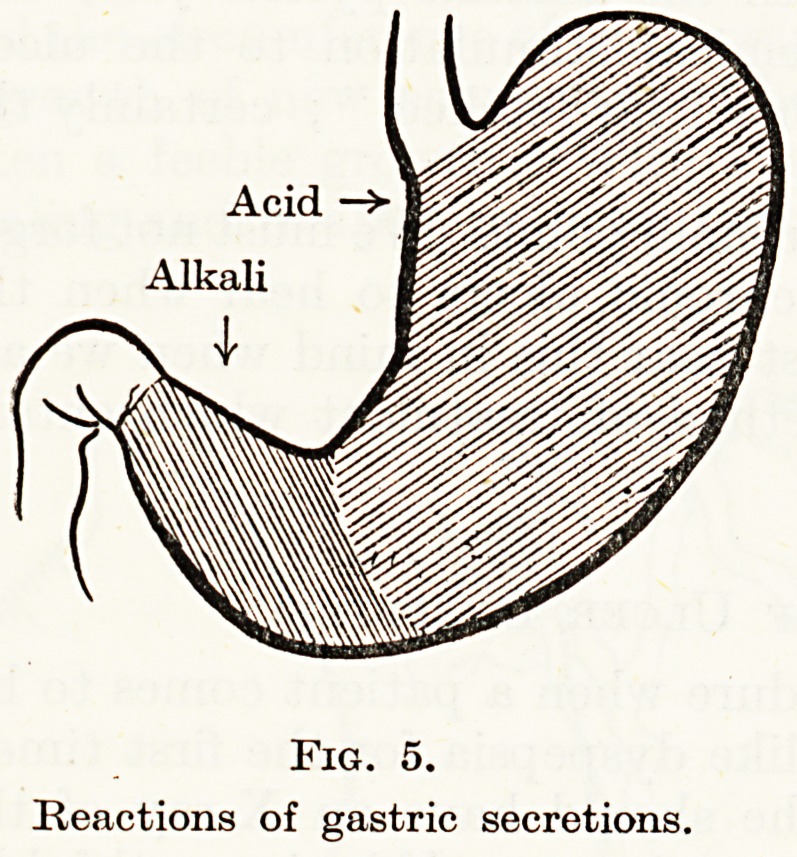


**Fig. 6. f6:**
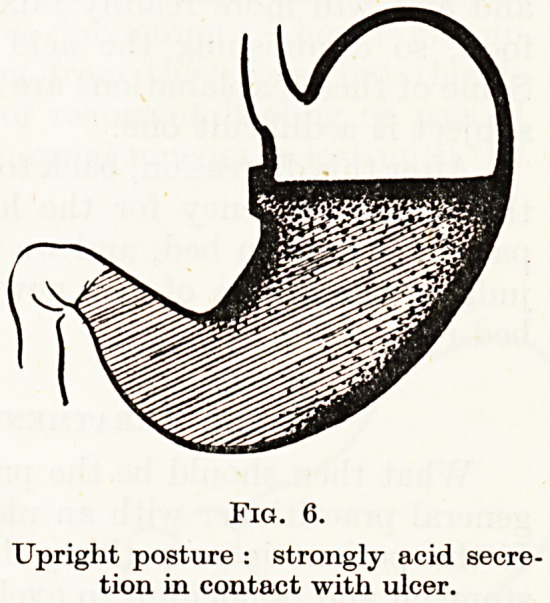


**Fig. 7. f7:**
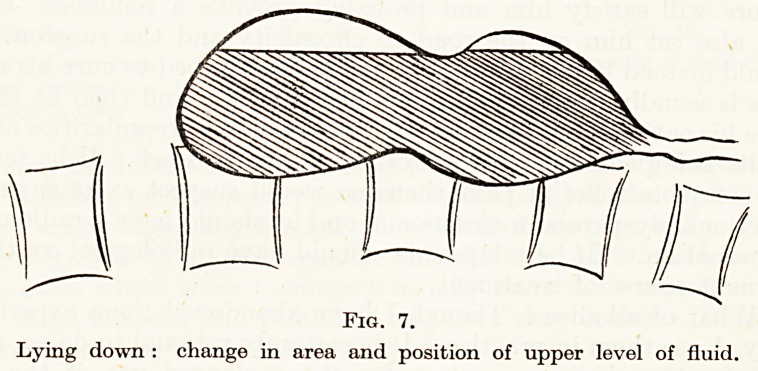


**Fig. 8. f8:**
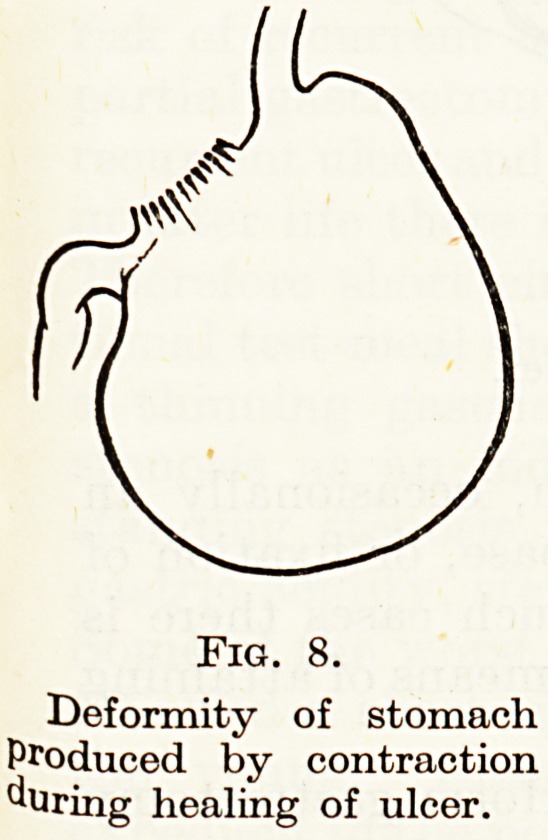


**Fig. 9. f9:**
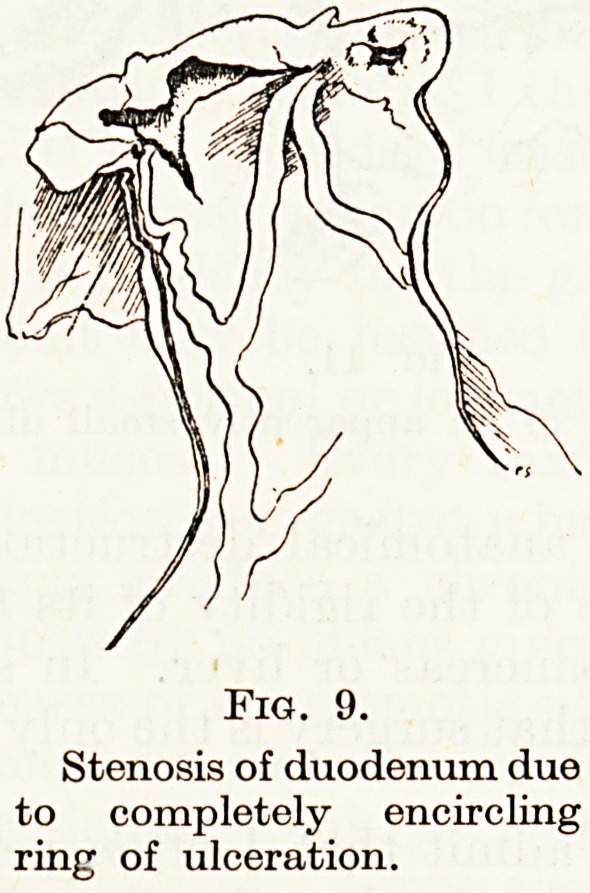


**Fig. 10. f10:**
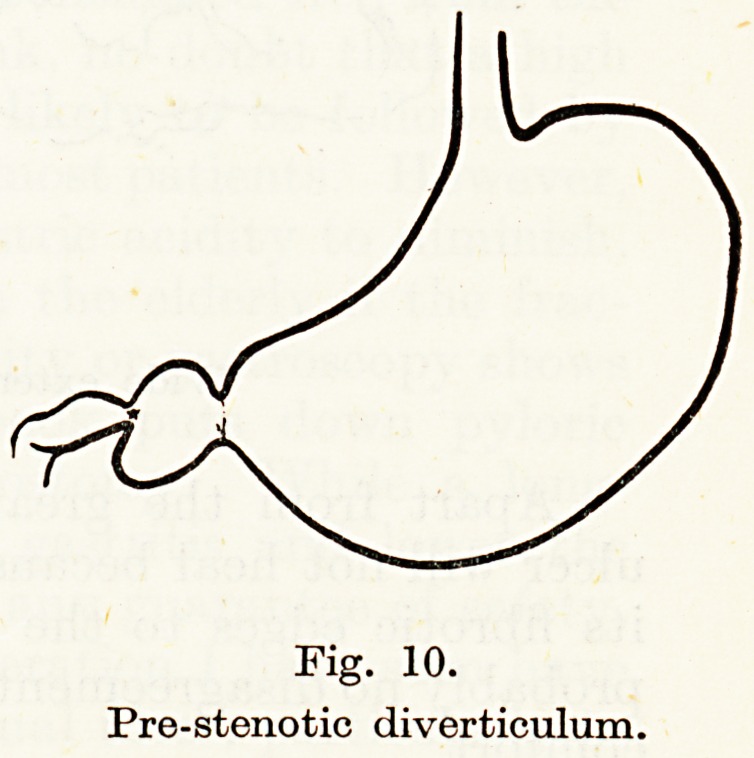


**Fig. 11. f11:**
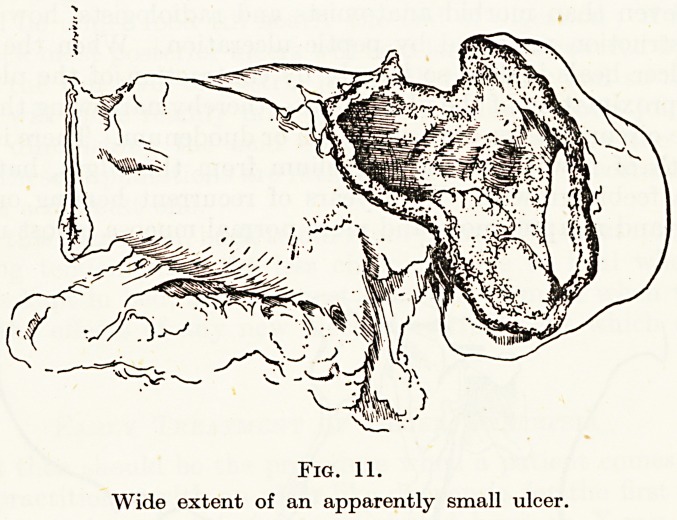


**Fig. 12. f12:**
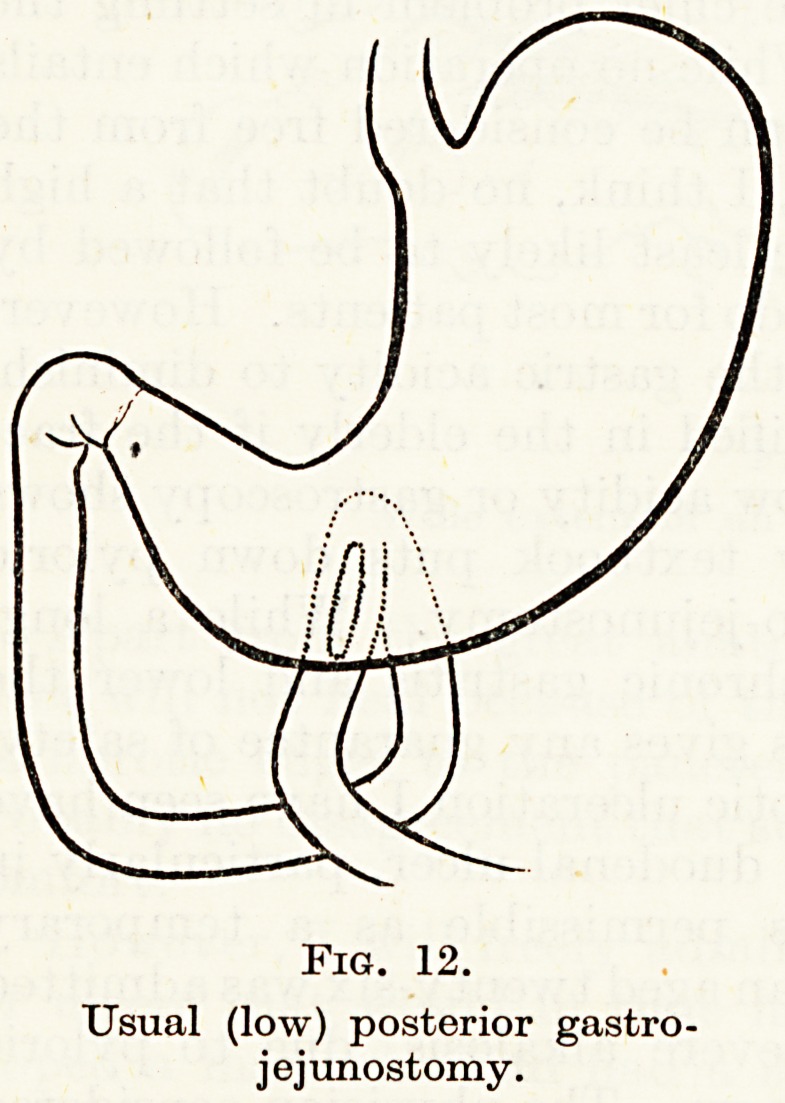


**Fig. 13. f13:**
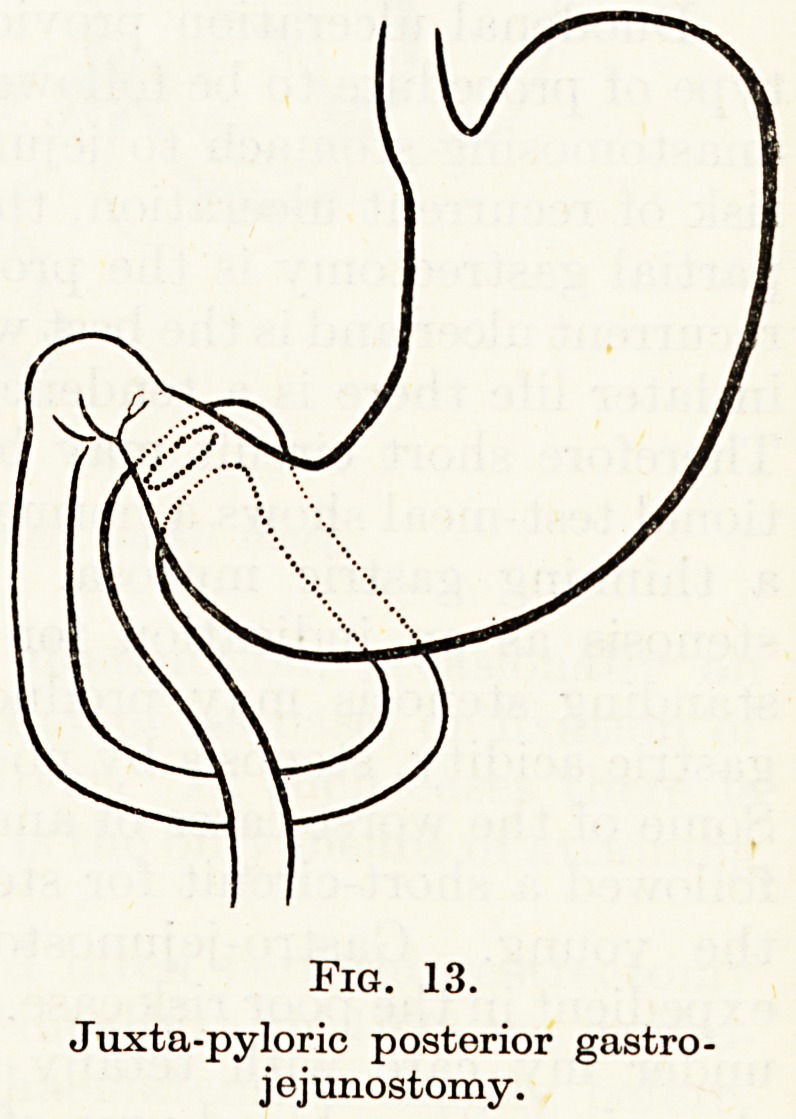


**Fig. 14. f14:**
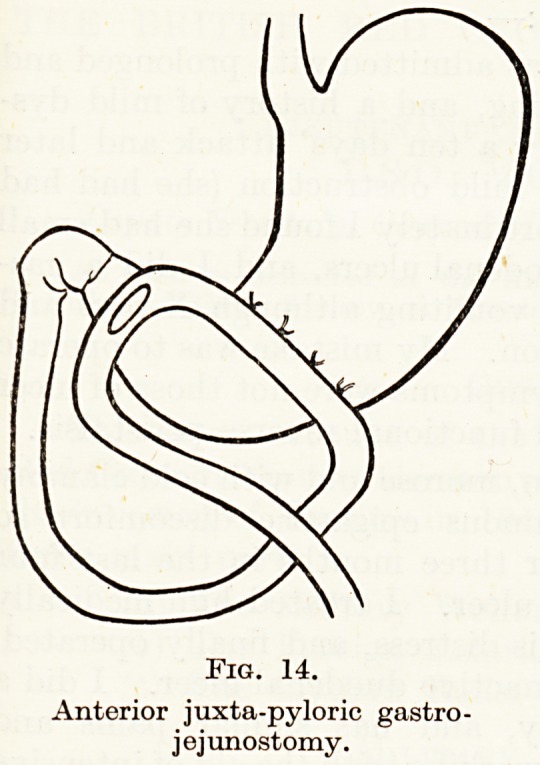


**Fig. 15. f15:**